# Risk predictive tools of perioperative drug hypersensitivity reaction: A case-control study

**DOI:** 10.1371/journal.pone.0262362

**Published:** 2022-01-13

**Authors:** Ujal Pradhan, Maliwan Oofuvong, Orarat Karnjanawanichkul, Jatuporn Pakpirom

**Affiliations:** Faculty of Medicine, Department of Anesthesiology, Prince of Songkla University, Hat Yai, Thailand; Boston Children’s Hospital, UNITED STATES

## Abstract

**Objective:**

We aimed to determine the risk factors of perioperative drug hypersensitivity reaction (DHR) and develop a predictive score for use in clinical practice.

**Methods:**

A case-control study was conducted in patients who underwent anesthesia at a tertiary hospital in Thailand, between 2015–2018. DHR cases were graded clinically from 1 to 4 according to the World Federation of Societies of Anesthesiologists by two anesthesiologists. Controls were randomly matched with cases (ratio 2:1) by age group and month and type of surgery. Patient and anesthesia-related factors and agents given intraoperatively were recorded. A risk score was derived from the coefficients of the significant predictors of the final multivariate logistic regression model. Risk scores, adjusted odds ratios (OR) for perioperative DHR and 95% confidence intervals (CI) were determined.

**Results:**

Overall, 325 cases and 650 controls were recruited. The severity of DHR was grade 1 (72.9%), grade 2 (24%), and grade 3 (3.1%). Our risk predictive tools for perioperative DHR provided a sensitivity of 62% and specificity of 65%. Predictive scores of subgroups of moderate to severe DHR showed high specificity (80%) but low sensitivity (47%). Common predictors of overall DHR and moderate to severe DHR were history of drug allergy to 2 or more drug categories (score 2.5–3.5), being allergic to analgesics (score 2.5–4.0), and intraoperative morphine use (score of 1). The sole predictor of high-risk perioperative DHR (score ≥3.5) was airway management with an endotracheal tube intubation (OR 5.6, 95% CI 2.2–14.4) whereas history of allergic rhinitis (OR 11.7, 95% CI 1.3–105.1) was a predictor of high-risk moderate to severe DHR (score ≥2.5).

**Conclusions:**

Our predictive tool for perioperative DHR provided a modest predictive ability. History of drug allergies, rhinitis, morphine use and endotracheal intubation were significant risk factors of DHR after adjusting for age and type of surgery.

## Introduction

Drug hypersensitivity reaction (DHR) is described as a mild to life-threatening reaction. Hypersensitivity reactions are reproducible signs or symptoms initiated by a drug at a dose tolerated by normal subjects [1]. The incidence of immediate allergic reactions during general anesthesia ranges from 1 in 20,000 to 1 in 10,000 and the mortality associated with immediate hypersensitivity reactions during anesthesia varies from 3% to 9%, depending on the country [2]. According to The Thai Anesthesia Incidents Study (THAI) study conducted between February 1, 2003, and January 31, 2004, in 20 hospitals across Thailand found that reaction-incidence was approximately 1 in 5,500 cases of anesthesia [3]. Known risk factors are age (elderly or infants) and age-related factors, having an underlying disease such as asthma, allergic rhinitis, hypertension, depression, or receiving concurrent medications such as angiotensin converting enzyme inhibitors and angiotensin receptor blockers [4–7].

In our institute, only severe DHR cases are referred for postoperative assessment by an allergist; however, some laboratory tests such as serum tryptase, and some skin allergy tests are still limited. The clinical symptoms used to diagnose perioperative DHR are still important for clinical management perioperatively. Moreover, risk factors originating from an analytic study between cases of perioperative DHR and matched controls are scarce. Therefore, our aim is to identify risk factors of DHR and develop a predictive tool that can assign risk scores perioperatively under the setting of a control-based study.

## Materials and methods

A retrospective case-control study was conducted at the Department of Anesthesiology, Songklanagarind Hospital, Prince of Songkla University between February 2015 and December 2018. The study (Protocol # 6123784) was approved by the Human Research Ethics Committee, Faculty of Medicine, Prince of Songkla University in full compliance with international guidelines for the Declaration of Helsinki (Chairperson Assoc. Prof. Boonsin Tangtrakulwanich) on 26 November 2018. The source of medical records and anesthetic records were from the hospital information system of Songklanagarind Hospital. All data were fully anonymized before accessed by the investigators. All patients who underwent anesthesia between February 2015 and December 2018 were eligible. Since it was a retrospective review, the ethics committee waived the need for informed consent. Patients who developed local anesthetic systemic toxicity, having blood product reaction, received monitored anesthetic care and had an American Society of Anesthesiologists (ASA) physical status 5 were excluded.

### Predictors and potential confounding variables

Patient-related, surgery-related and anesthesia-related factors, except for the matching variables, were included as potential predictors in the modelling process. Patient-related factors were sex, concomitant disease, and history of allergy. History of drug allergy was referred to type B reactions (hypersensitivity reactions) and confirmed by an allergist. The details of assessment and symptoms were shown in the hospital information system. Surgery-related factors were type of case (outpatient, elective, emergency), and estimated blood loss at the time of the DHR event. Anesthesia-related factors were ASA physical status, type of anesthesia, airway management, duration of anesthesia and intraoperative anesthetic agents.

### Outcome of the study

Cases were defined as patients who had clinical symptoms of hypersensitivity reaction intraoperatively and at the post-anesthetic care unit. Cases were extracted from the hospital information system and reviewed by two nurse anesthetists and confirmed by two anesthesiologists (UP and MO). To ensure reasonably accurate classification of the cases, period of anesthesia when DHR occurred and time to first DHR event were recorded. Time to first DHR was calculated from starting time of anesthesia to DHR occurrence. Nurse anesthetists recorded all clinical symptoms related with DHR (rash, bronchospasm, hypotension) at 0–30 minutes after first symptoms of DHR occurring. Periods of anesthesia were divided into induction (first 10 minutes after starting anesthesia), maintenance (10 minutes after starting anesthesia until skin closure), and emergence period (after skin closure until the end of anesthesia).

After DHR cases were identified, the grading of DHR was characterized according to grading of anaphylaxis of the World Federation of Societies of Anesthesiologists [6]. Mild DHR (grade 1) was defined as only generalised cutaneous signs (erythema, urticaria, pruritus, angioedema) developed after exposure to the agent. Moderate DHR (grade 2) was defined as the presence of cutaneous signs and moderate multiorgan (respiratory, cardiovascular) involvement with cutaneous signs. Severe DHR was defined as the presence of severe life-threatening multiorgan involvement (grade 3) or circulatory or respiratory arrest (grade 4). Controls were randomly selected among patients who did not develop a drug hypersensitivity reaction and matched with cases in a ratio of 2:1 based on month of surgery, type of surgery, and age group (0–1, 2–7, 8–18, 19–65, >65 years).

### Definition of clinical symptoms related with DHR

Erythema was defined as superficial reddening of the skin, usually occurring in patches, and usually due to irritation. Urticaria was defined as an outbreak of swollen, pale red bumps or plaques (wheals) on the skin. Pruritus was defined as an unpleasant sensation of the skin that provokes the urge to scratch. Angioedema was defined as an area of swelling of the lower layer of skin and tissue just under the skin or mucous membranes. The swelling may occur in the face, tongue, larynx, abdomen, or arms and legs. Desaturation was defined as a condition in which there is low blood oxygen leading to a pulse oximetry reading less than 92%. Cyanosis was defined as a bluish discoloration of the skin. Bronchospasm/wheezing was defined as spasm of the airway or narrowing of the airway diameter leading to a high-pitched whistling sound when breathing. Tachycardia was defined as an increased heart rate of more than 100 beats per minute in adults or more than 10% of baseline level in children. Bradycardia was defined as a decrease in heart rate below 60 beats per minute in adults or more than 10% of baseline level in children or requiring atropine to maintain a normal heart rate. Hypotension was defined as a decrease in mean arterial pressure (MAP) by ≥30% of baseline level. Severe life-threatening multiorgan involvement was defined as arrhythmia, cardiovascular collapse (decrease in MAP by >30% of baseline level for ≥ 3 minutes or requiring catecholamines to maintain normal blood pressure) with severe bronchospasm or generalized cutaneous signs.

### Statistical analysis

Data record forms were created and information was abstracted from the electronic medical records, then double-entered into a database using EpiData version 3.1. R software was used to analyze the data (R version 4.0.2, R Core Team, Vienna). All variables are presented descriptively with mean and standard deviation or median and interquartile range (IQR) as appropriate for continuous variables, and frequency and percentage for categorical variables. Post-hoc analysis was performed for multiple comparisons between groups when the overall differences were significant. Variables having a p-value ≤ 0.2 from the univariate analysis were included in the initial multivariate logistic regression model. The model was refined by sequential backward elimination of non-significant variables performed by the likelihood ratio test. The final model includes only significant factors and the strengths of their association with being a case presented as adjusted odds ratios (OR) with 95 confidence intervals (CI). Subgroup analyses of moderate to severe DHR was also performed using multivariate logistic regression in a similar manner. Factors were considered significant if their p-values were < 0.05.

### Risk predictive score

The risk predictive score of mid to severe DHR (case) was developed using the coefficients of the significant covariates in the final logistic regression model. Scores were obtained by multiplying each coefficient by 2 and then rounding the result to the nearest 0.5 or 1.0 numeric for higher accuracy. Model discrimination performance was examined using the area under the receiver operating characteristic (ROC) curve providing sensitivity and specificity based on the optimal cut-point of the risk score. The risk predictive score for the subgroup moderate to severe DHR was performed in a similar manner.

### Sample size determination

We estimated the prevalence of exposure (potential predictor) among the controls to be 10%, and with a ratio of controls to cases of 2:1 to detect an odds ratio of at least 2.0 using a significance level of 0.05 and a power of 90%, a sample size of at least 277 cases and 554 controls was required.

## Results

**Fig 1:**
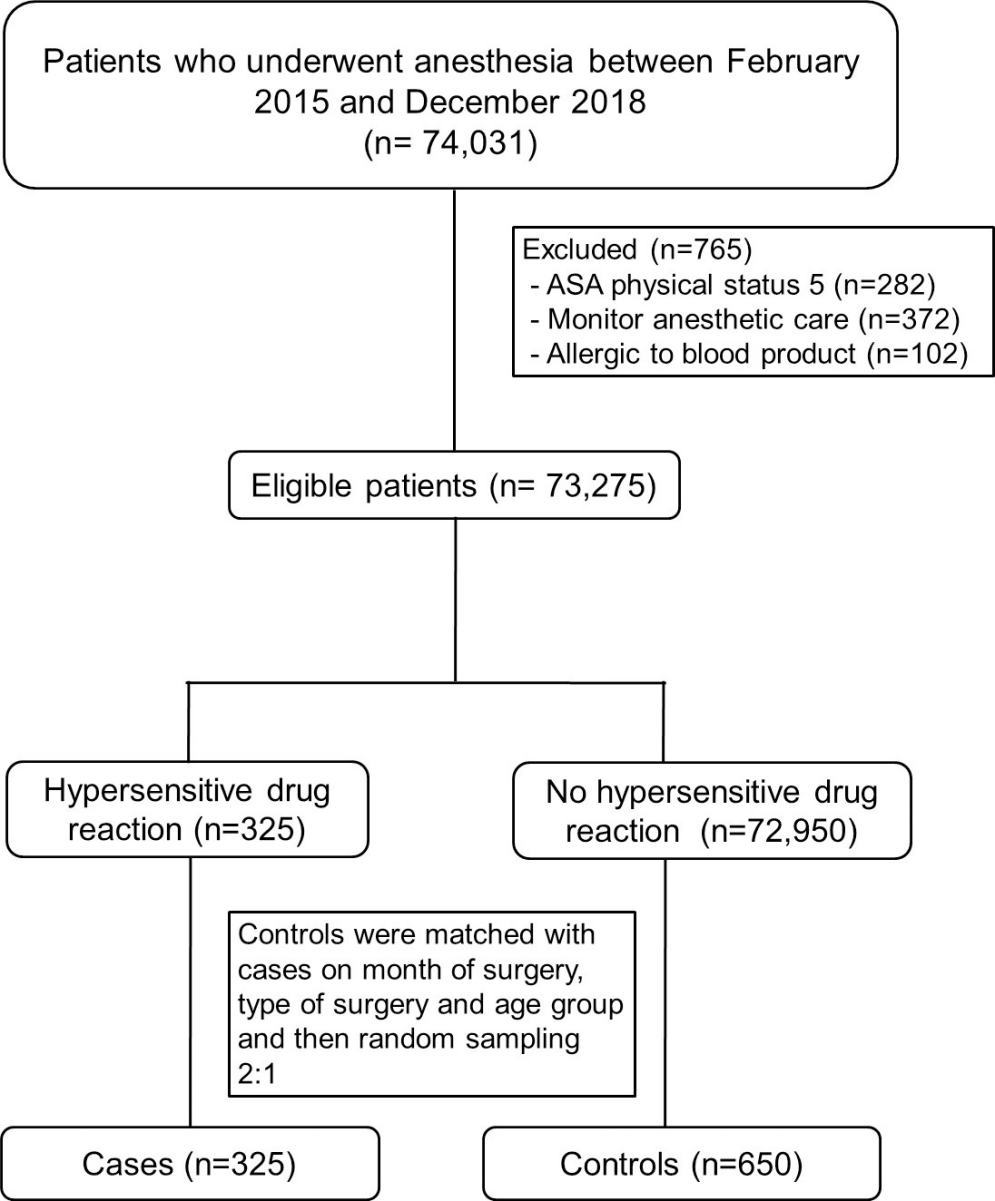
Flow diagram of the study. ASA, American Society of Anesthesiologists.

**Fig 2:**
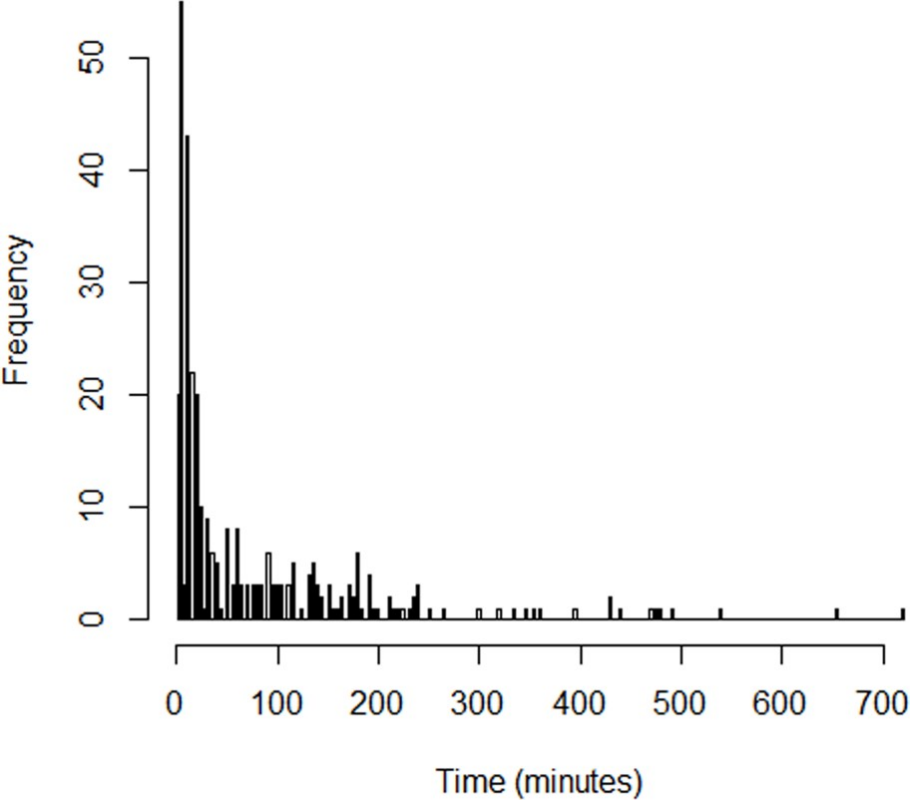
Histogram of time to first drug hypersensitivity reaction.

A total of 325 cases (237 mild, 78 moderate and 10 severe) and 650 controls were recruited (Fig 1). As shown in Table 1, the most common clinical symptom in all grades of DHR was urticaria (91%), which was mainly treated with chlorpheniramine 75%. Bronchospasm/wheezing occurred 6 of the 10 grade 3 patients whereas it was less common in patients with mild-moderate DHR. Hypotension occurred in all grade 3 patients (100%) whereas it occurred in 76% of grade 2 patients. As shown in Table 2, DHR events were more common during the induction period (35.5%). During the maintenance period, grade 2 (39.7%) and grade 3 patients (50%) were more common. The distribution of time to first DHR event (Fig 2) was heavily right-skewed with most events occurring within the first 10 minutes after induction. Table 3 compares the distribution of factors between cases and controls. Most variables were well balanced with only history of allergies, ASA physical status, and airway management showing a significant imbalance in the distributions. Although age group was used as a matching variable, actual age in years was significantly different between cases and controls (p = 0.013), therefore, we expanded the age group of 19–65 into two age groups of 19–44 and 45–65 and included new age group in the multivariate model to reduce the confounding. The distribution of anesthetic agents given intraoperatively among cases and controls are presented in Table 4. The agent categories, namely narcotic, neuromuscular blocking agent (NMBA), antibiotic, and inhalation agent, or individual agent such as ketorolac, glycopyrrolate and levobupivacaine, which had a p-value ≤ 0.2 on univariate analysis, were included in the initial multivariate model with other potential risk factors.

**Table 1:** Distribution of symptoms by severity of drug hypersensitivity reaction.

Symptoms and treatment of DHR	Grade 1 (n = 237)	Grade 2 (n = 78)	Grade 3 (n = 10)
**Skin Symptoms**			
Erythema	34 (14.3)	12 (15.4)	1 (10.0)
Pruritus	17 (7.2)	9 (11.5)	0 (0)
Urticaria	216 (91.1)	72 (92.3)	9 (90.0)
**Treatment**			
Chlorpheniramine	196 (82.7)	75 (96.2)	7 (70.0)
Ondansetron	1 (0.4)	2 (2.6)	0 (0)
**Angioedema**	3 (1.3)	16 (20.5)	7 (70.0)
**Treatment**			
Dexamethasone	3 (1.3)	16 (20.5)	7 (70.0)
Adrenaline	1 (0.4)	9 (11.5)	5 (50.0)
**Respiratory Symptoms**			
Desaturation (SpO2 <92%)	0 (0)	3 (3.8)	3 (30.0)
Cyanosis	0 (0)	1 (1.3)	2 (20.0)
Bronchospasm/wheezing	0 (0)	17 (21.8)	6 (60.0)
**Treatment**			
Salbutamol	1 (0.4)	15 (19.2)	7 (70.0)
**Cardiovascular Symptoms**			
Tachycardia	0 (0)	1 (1.3)	0 (0)
Bradycardia	0 (0)	6 (7.7)	0 (0)
Hypotension	0 (0)	59 (75.6)	10 (100)
**Treatment**			
**Vasopressors**			
Norepinephrine	5 (2.1)	18 (23.1)	10 (100)
Adrenaline	0 (0)	2 (2.6)	5 (50)
Ephedrine	5 (2.1)	44 (56.4)	4 (40)
**Crystalloids**			
Lactated ringer’s solution	0 (0)	7 (9.0)	1 (10)
Normal saline	0 (0)	4 (5.1)	2 (20)
**Colloids**			
Voluven^®^	0 (0)	2 (2.6)	1 (10)
Gelofusine^®^	0 (0)	72 (92.3)	9 (90)

The values are presented as the number of patients (%) per group, DHR, drug hypersensitivity reaction.

**Table 2:** Distribution of events based on severity and time from induction to first drug hypersensitivity reaction by period (N = 325).

Period	Time from induction to first event (min)	Grade 1 (n = 237)	Grade 2 (n = 78)	Grade 3 (n = 10)	p-value
Induction	5 [5, 10]	90 (38.0)	30 (38.5)	2 (20)	0.507
Maintenance	25 [20, 50]	62 (26.2) ^a^	31 (39.7) ^b^	5 (50) ^ab^	0.029*
Emergence	105 [60, 201]	33 (14.3)	12 (15.4)	3 (30)	0.398
PACU	140 [90, 180]	52 (21.9) ^a^	5 (6.4) ^b^	0 (0) ^ab^	0.003*

**Note**: The values are presented as the median [interquartile range], or the number of patients (%) per group,

*p<0.05 by Fisher’s exact test, ^ab^ Groups sharing the same superscript were not significantly different. PACU, post anesthesia care unit.

**Table 3:** Comparison of patient, surgical and anesthesia-related factors of mild to severe drug hypersensitivity reaction.

Variable	Case (n = 325)	Control (n = 650)	p-value
**Patient-related factors**			
Age (years)	37 [25, 49]	41 [30, 53]	0.013*
Age group, years: 0-1/1-7/8-18/19-65/>65	1/8/30/261/25	2/16/60/522/50	Match
Male	105 (32.3)	234 (36.0)	0.285
Case			0.213
OPD	6 (1.8)	6 (0.9)	
Elective	250 (76.9)	481 (74.0)	
Emergency	69 (21.2)	163 (25.1)	
Concomitant disease			
Asthma	4 (1.2)	6 (0.9)	0.739
Allergic Rhinitis	17 (5.2)	14 (2.2)	0.017**
COPD	0 (0)	1 (0.2)	1
Hypertension	41 (12.6)	109 (16.8)	0.109
Drugs allergy	55 (16.9)	59 (9.1)	<0.001**
At least 2 categories	16 (4.9)	14 (2.2)	0.002***
ATB	15 (4.6)	22 (3.4)	
Analgesic	16 (4.9)	11 (1.7)	
Others	3 (0.9)	9 (1.4)	
Unknown	5 (1.5)	3 (0.5)	
Foods allergy	22 (6.8)	20 (3.1)	0.018**
Seafood	20 (6.2)	17 (2.6)	0.024***
Daily product	1 (0.3)	2 (0.3)	
Alcohol	1 (0.3)	1 (0.2)	
Others allergy	0 (0)	4 (0.6)	0.308
**Surgical and anesthesia-related factors**			
Type of Surgery			Match
Cardiac/Obgyn/Ortho/Neuro/GU/ENT/Vascular/Plastic/Abdomen/Thoracic/Remote/Eye	4/107/80/10/10/12/7/4/75/7/5/4	8/214/160/20/20/24/14/8/150/14/10**/**8	
ASA physical status			0.063
1	37(11.4)	59 (9.1)	
2	241 (74.2)	454 (69.8)	
3	41 (12.6)	125 (19.2)	
4	6 (1.8)	12 (1.8)	
Type of anesthesia			0.668
GA only	205 (63.1)	391 (60.2)	
RA only	66 (20.3)	140 (21.5)	
Combined GA and RA	54 (16.6)	119 (18.3)	
Airway management			0.051
Room air	66 (20.3)	138 (21.2)	
Oxygen insufflation	8 (2.5)	29 (4.5)	
Mask	0 (0)	5 (0.8)	
Laryngeal mask airway	17 (5.2)	52 (8.0)	
Endotracheal tube intubation	234 (72.0)	426 (65.5)	
Type of RA			0.969
Spinal block only	64 (53.3)	135 (52.1)	
PNB only	39 (32.5)	90 (34.7)	
Epidural block only	9 (7.5)	17 (6.6)	
Combined spinal and PNB	8 (6.7)	17 (6.6)	
Duration of anesthesia (min)	145 [90, 230]	135 [85, 210]	0.095
Duration of anesthesia ≥2 hours	208 (64.0)	383 (58.9)	0.144
EBL at the time of symptom (ml)	100 [20, 300]	100 [20, 300]	0.453

**Note**: The values are presented as the median [interquartile range], or the number of patients (%) per group, *p<0.05 by Wilcoxon ranksum test,

**p<0.05 by Chi-square test,

***p<0.05 by Fisher’s exact test, remote = cardiac catheterization/X-ray/gastrointestinal scope.

OPD, outpatient department; COPD, chronic obstructive pulmonary disease; ATB, antibiotics; Obgyn, obstetrics & gynecology; Ortho, orthopedics; Neuro, neurology; GU, genitourinary; ENT, ear-nose-throat; ASA, American Society of Anesthesiologists; GA, general anesthesia; RA, regional anesthesia; PNB, peripheral nerve block; EBL, estimated blood loss.

**Table 4:** Distribution of anesthetic agents receiving during intraoperative period among cases with mild to severe drug hypersensitivity reaction and controls (N = 975).

Category	Case (n = 325)	Control (n = 650)	p-value
**Intravenous induction agents**	**261 (80.3)**	**508 (78.2)**	0.488
Propofol	257 (79.1)	496 (76.3)	0.373
Thiopental	2 (0.6)	2 (0.3)	0.604
Etomidate	0 (0)	4 (0.6)	0.308
Ketamine	5 (1.5)	14 (2.2)	0.682
**Narcotics**	**302 (92.9)**	**579 (89.1)**	0.070
Fentanyl	201 (61.9)	429 (66.0)	0.227
Morphine	165 (50.8)	238 (36.6)	<0.001*
Pethidine	23 (7.1)	15 (2.3)	<0.001*
**Neuromuscular blocking agents**	**235 (72.3)**	**428 (65.9)**	0.049*
Cisatracurium	170 (52.3)	342 (52.6)	0.982
Rocuronium	57 (17.5)	82 (12.6)	0.048*
Succinylcholine	45 (13.9)	51 (7.9)	0.004*
**Sedative agents**	**82 (25.2)**	**141 (21.7)**	0.246
Midazolam	80 (24.6)	139 (21.4)	0.290
Dexmedetomidine	2 (0.6)	4 (0.6)	0.999
**Inhalation anesthetic agents**	**212 (65.2)**	**454 (69.9)**	0.165
Sevoflurane	191 (58.8)	414 (63.7)	0.155
Desflurane	22 (6.8)	43 (6.6)	0.999
**Antibiotics**	**289 (88.9)**	**539 (82.9)**	0.018*
Ampicillin	22 (6.8)	46 (7.1)	0.965
Cefazolin	201 (61.9)	376 (57.9)	0.259
Ceftriazone	48 (14.8)	74 (11.4)	0.161
Metronidazone	41 (12.6)	64 (9.9)	0.228
Clindamycin	11 (3.3)	12 (1.9)	0.205
Other^†^	16 (4.9)	36 (5.5)	0.960
**NSAIDs**	**38 (11.7)**	**60 (9.2)**	0.275
Ketorolac	9 (2.8)	8 (1.2)	0.141
Dynastat	29 (8.9)	52 (0.1)	0.712
**Reversal agents**	**198 (60.9)**	**370 (56.9)**	0.261
Neostagmine	196 (60.3)	369 (56.8)	0.324
Suggamadex	2 (0.6)	2 (0.3)	0.604
**Anticholinergic**	**196 (60.3)**	**367 (56.5)**	0.262
Atropine	194 (59.7)	367 (56.5)	0.372
Glycopyrrolate	2 (0.6)	0 (0)	0.110
**Regional anesthesia**	**121 (37.2)**	**241 (37.1)**	0.999
Lidocaine	14 (4.3)	20 (3.1)	0.422
Hyperbaric bupivacaine	65 (0.2)	145 (22.3)	0.457
Isobaric bupivacaine	50 (15.4)	85 (13.1)	0.376
Levobupivacine	2 (0.6)	17 (2.6)	0.060

**Note**: The values are presented as the number of patients (%) per group. *p<0.05 by Chi-square test.

^**†**^Others include gentamycin, ciprofloxacin, penicillin, cloxacillin, co-amoxiclav and imipenem.

NSAIDs, Nonsteroidal anti-inflammatory drugs.

### Logistic regression predicting DHR and risk score of perioperative DHR

**Fig 3:**
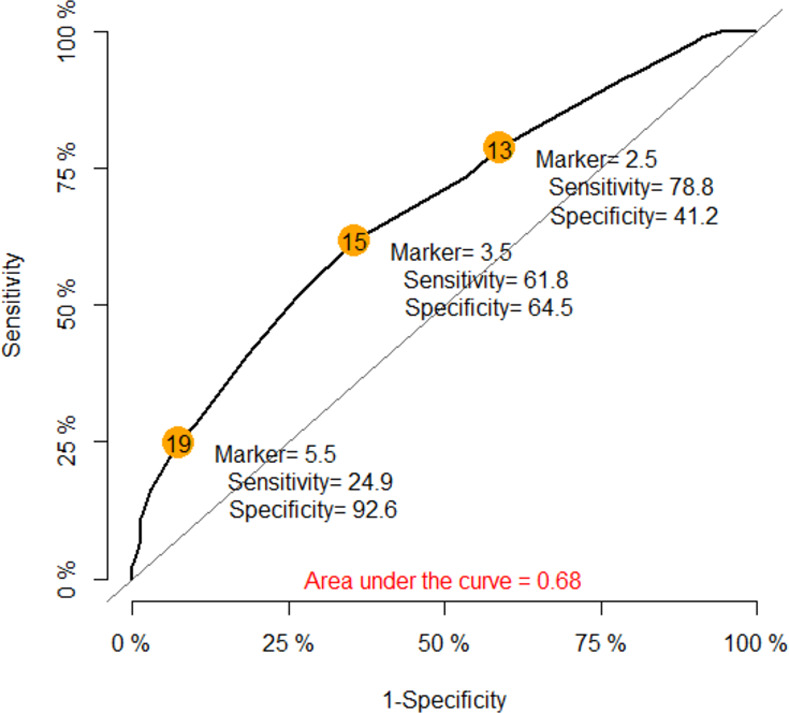
Predictive ability of final logistic model for perioperative drug hypersensitivity reaction.

Twenty-four variables, including 5 patient-related factors (age group, history of allergic rhinitis, food allergy, drug allergy, hypertension), 3 surgical- and anesthesia-related factors (ASA physical status, airway management and duration of anesthesia) and 16 anesthetic agents were included in the initial multivariate model. The results and risk scores from the model are shown in Table 5. We selected the reference group of each factor based on the subgroup which had the lowest risk for DHR except for use of anesthetic agents in which all the reference groups were no use. The scores were summed to obtain an individual risk score which ranged from -5 to 10. Fig 3 shows the ROC curve of the individual risk scores predicting DHR. The area under the curve was 0.68 and the optimal cut-point based on the highest summation of sensitivity (62%) and specificity (65%) of the model was 3.5. The risk scores were then classified into three groups: high (≥ 3.5), intermediate (1–3), and low (≤ 0) that indicated the risk level of having mid to severe perioperative DHR. Predictors of high risk DHR (score ≥3.5) was airway management with an endotracheal tube intubation (vs oxygen insufflation) (OR 5.6, 95% CI 2.2–14.4). Intermediate risk factors (score 1–3) were history of seafood allergy (OR 2.7, 95% CI 1.3–5.5), history of drug allergy to multiple drug categories (OR 3.7, 95% CI 1.7–8.0) and being allergic to analgesics (OR 3.7, 95% CI 1.7–8.9), ASA physical status 1 (vs 3) (OR 2.2, 95% CI 1.2–4.1), intraoperative opioids use (morphine [OR 1.6, 95% CI 1.2–2.2] and pethidine [OR 4.4, 95% CI 2.2–9.0]), succinylcholine use (OR 1.9, 95% CI 1.2–3.0) and antibiotics use (cefazolin [OR 1.5, 95% CI 1.04–2.0] and ceftriaxone [OR 1.7, 95% CI 1.03–2.7]). Agents found to decrease the risk of DHR were sevoflurane use (risk score of -2.5), desflurane use (risk score of -2.0) and levobupivacaine use (risk score of -3.5). One case of DHR was allergic to ceftriazone and oliclinomel but received ceftriazone intraoperatively. The patient developed cutaneous symptoms (mild DHR) only.

**Table 5:** Multivariate logistic regression model of predictors and risk scores of perioperative drug hypersensitivity reaction.

Variable	Coefficient	Adjusted OR (95% CI)	p-value	Risk score
**Age group (years) (Ref = 45–65)**				
0–7	0.90	2.45 (0.96, 6.30)	0.062	0
8–18	0.63	1.87 (1.07, 3.26)	0.028	1.5
19–44	0.46	1.58 (1.11, 2.24)	0.011	1.0
>65	0.67	1.96 (1.08, 3.57)	0.027	1.5
**History of rhinitis**	0.73	2.07 (0.92, 4.70)	0.080	0
**Food allergy (Ref = None)**				
Seafood	0.98	2.66 (1.28, 5.51)	0.009	2.0
Dairy products/Alcohol	0.34	1.40 (0.20, 9.87)	0.734	0
**Drug allergy (Ref = No)**				
Antibiotics	0.57	1.77 (0.85, 3.70)	0.126	0
Analgesics	1.34	3.82 (1.65, 8.86)	0.002	2.5
Multiple	1.30	3.68 (1.68, 8.03)	0.001	2.5
Other/unknown	0.39	1.48 (0.57, 3.86)	0.421	0
**ASA physical status (Ref = 3)**				
1	0.79	2.20 (1.19, 4.09)	0.013	1.5
2	0.40	1.43 (0.92, 2.24)	0.115	0
4	0.20	1.22 (0.34, 4.40)	0.761	0
**Airway management (Ref = Mask/Oxygen insufflation)**				
Room air	0.36	1.43 (0.59, 3.50)	0.432	0
Laryngeal mask airway	0.86	2.36 (0.82, 6.78)	0.110	0
Endotracheal tube intubation	1.72	5.59 (2.18, 14.38)	<0.001	3.5
**Use of anesthetic agents**				
Morphine	0.46	1.58 (1.16, 2.16)	0.004	1.0
Pethidine	1.48	4.38 (2.14, 8.96)	<0.001	3.0
Succinylcholine	0.63	1.87 (1.16, 3.01)	0.010	1.5
Sevoflurane	-1.26	0.29 (0.17, 0.48)	<0.001	-2.5
Desflurane	-1.06	0.35 (0.16, 0.74)	0.006	-2.0
Lidocaine for PNB	0.74	2.10 (0.97, 4.55)	0.060	0
Levobupivacaine for PNB	-1.65	0.19 (0.04, 0.91)	0.038	-3.5
Cefazolin	0.38	1.46 (1.04, 2.04)	0.030	1.0
Ceftriaxone	0.52	1.68 (1.03, 2.72)	0.037	1.0

**Note**: p value by Wald test.

ASA, American Society of Anesthesiologists; OR: Odds ratio; CI, confident interval; Ref, reference group; PNB, peripheral nerve block.

### Subgroup analysis of moderate to severe DHR

**Fig 4:**
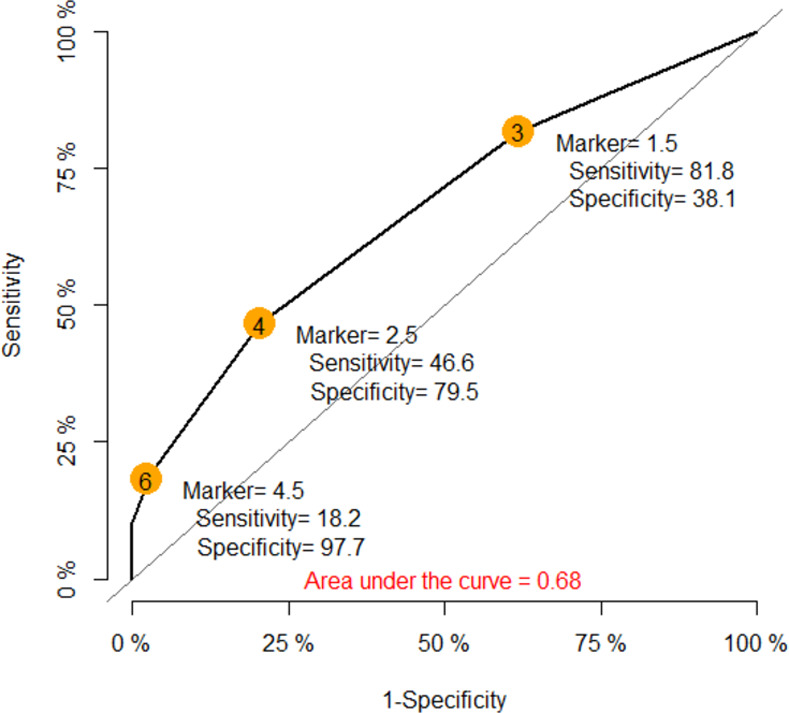
Predictive ability of final logistic model for moderate to severe drug hypersensitivity reaction.

S1 and S2 Tables compare the distribution of factors and anesthetic agents between moderate to severe DHR and controls. Eight variables, including 2 patient-related factors (history of allergic rhinitis and drug allergy), 1 anesthesia-related factor (duration of anesthesia) and 5 anesthetic agents (morphine, clindamycin, lidocaine, isobaric bupivacaine and levobupivacaine) were included in the initial multivariate model. Age was not significantly different between moderate to severe DHR and controls (p = 0.5), therefore, a new age group category was not created nor included in the multivariate model. Risk scores from the model are shown in Table 6. The scores ranged from 0 to 11.5. Fig 4 shows the ROC curve of the individual risk scores predicting moderate to severe DHR. The area under the curve was 0.68 and the optimal cut-point based on the highest summation of sensitivity (47%) and specificity (80%) of the model was 2.5. The risk scores were then classified into three groups: high (≥ 2.5), intermediate (1–2), and low (≤ 0) that indicated the risk level of having moderate to severe perioperative DHR. Predictors of high-risk moderate to severe DHR (score ≥2.5) were history of allergic rhinitis (OR 11.7, 95% CI 1.3–105.1), history of drug allergy to 2 or more drug categories (OR 6.3, 95% CI 1.2–33.1) and being allergic to analgesics (OR 8.1, 95% CI 1.3–48.5). Intermediate risk factors (score 1–2) were duration of anesthesia ≥2 hours (OR 2.3, 95% CI 1.2–4.4) and intraoperative morphine use (OR 1.8, 95% CI 1.03–3.2).

**Table 6:** Subgroup analysis for predictors and risk scores of moderate to severe drug hypersensitivity reaction (cases = 88, controls = 176).

Variable	Coefficient	Adjusted OR (95% CI)	p-value	Risk score
**History of rhinitis**	2.46	11.68 (1.30, 105.1)	0.028	5.0
**Drug allergy (Ref = None)**				
Antibiotics	1.30	3.66 (0.75, 17.85)	0.108	0
Analgesic	2.09	8.06 (1.34, 48.51)	0.022	4.0
Multiple	1.84	6.28 (1.19, 33.1)	0.030	3.5
Other/unknown	0.86	2.37 (0.49, 11.38)	0.280	0
**Duration of anesthesia (Ref = <2 hours) ≥2 hours**	0.85	2.34 (1.24, 4.42)	0.009	1.5
**Morphine use**	0.60	1.83 (1.03, 3.24)	0.038	1.0

**Note**: p-value by Wald test.

OR, odds ratio; CI, confident interval; Ref, reference group.

## Discussion

We included 325 DHR cases, of which 73% were mild and 27% were moderate to severe, having multiorgan involvement and hemodynamic instability after exposure to suspected agents. Since only severe DHR cases are referred for an allergist assessment for skin test, and serum tryptase could not be performed in our institute, we constructed a risk predictive tool for perioperative DHR which provided a modest predictive ability (sensitivity of 62% and specificity of 65%). We also constructed a risk predictive tool for moderate to severe DHR which provided low sensitivity (47%) and high specificity (80%) which could be specific to high-risk group of moderate to severe DHR cases. The severity (Table 1) and timing (Fig 2) of DHR during the induction phase was common and supported by other studies [8–10]. This is likely due to the simultaneous use of different combinations of drugs for induction (narcotics, propofol and NMBA) before surgery commenced. A study from the United States showed that DHR was more common during the maintenance period [11] whereas this period was the most common period of moderate to severe DHR occurrence in our study.

### Common risk factors for perioperative and moderate to severe DHR

Drug allergy was a common risk factor for both perioperative and moderate to severe DHR in our study, a result similar to a study by Dewachter et al [12]. Allergy to at least 2 categories of drugs was an intermediate risk factor of perioperative DHR (risk score of 2.5) but was a high risk of moderate to severe DHR in our study (risk score of 3.5). It is also known as multiple drug allergy syndrome due to cross reactivity between the different categories of drugs [12,13]. This cross reactivity could be drug-drug or drug-food. Therefore, anesthesiologists should reduce the risk of DHR by being aware of a patient’s known drug allergies, especially if the patient has an allergy to analgesics (risk score of 4.0). Our hospital has had a well-designed hospital information system since 2015. It can alert the physician via a pop-up message which is triggered if the physician attempts to prescribe a drug to which the patient is known to be allergic. During the study period, only 1 patient received a drug to which they had a known drug allergy.

Morphine was also a common risk factor, having an intermediate risk (risk score = 1) for both perioperative DHR and moderate to severe DHR from the risk predictive tool. The incidence of DHR due to opioids ranges from 1 in 100,000 to 1 in 200,000 [14]. Morphine is known to cause histamine release and cause cutaneous symptoms, which is rarely IgE-mediated hypersensitivity, but mostly forms a non-IgE-mediated anaphylactoid reaction involving complementary opioid receptors causing histamine release leading to the cutaneous symptoms. Non-allergic histamine reactions are much more prevalent than IgE-mediated hypersensitivity to opioids such as morphine [15,16]. In our institute, we tend to use morphine in healthier patients, for example those with ASA physical status 1, or as an additive, for example intrathecal morphine for spinal anesthesia.

### Risk predictive tool for perioperative DHR

#### High risk group (risk score ≥3.5)

In our study airway management with an endotracheal tube, when compared to oxygen insufflation, had the highest risk of DHR. This may be because during intubation procedures, different types of anesthetic agents can be used. Common drugs that cause DHR are NMBAs, which are commonly used during endotracheal tube intubation, whereas oxygen insufflation avoids the need to use these drugs. Our study found non-depolarized NMBA (cisatracurium, vecuronium) were not significant predictors and were removed from the final model. However, succinylcholine use was an intermediate risk for DHR in our study which was supported by many studies [17–19]. Therefore, if succinylcholine (risk score of 1.5) was used for endotracheal intubation (risk score of 3.5), the combined risk score would increase and cause the patient to fall into the high-risk group. Moreover, DHR can be caused by insertion of the endotracheal tube itself, especially if the tube is made of silicone. In such cases, use of an endotracheal tube made of polyvinyl would be preferred [20]. In our institute, we only use polyvinyl endotracheal tubes, therefore the high-risk score must arise from combined anesthetic agents.

### Intermediate risk group (risk score 1–3)

#### Age

The elderly (age >65 years) and those aged 8–18 years had an intermediate risk of developing perioperative DHR (risk score of 1.5). This finding was supported by other studies which showed that increasing age increased the risk of DHR [4,8]. Harper et.al [8] found that increasing age was associated with increased severity and fatality of DHR. However, we could not find evidence to support age 8–18 years having an increased risk of DHR.

#### ASA physical status

According to our finding, ASA physical status 1 had a higher risk of DHR when compared to ASA 3, in contrast to other studies [8,9]. This may be because we often administered morphine in healthy patients (ASA 1) which might cause cutaneous symptoms from non-IgE-mediated anaphylactoid reaction/histamine release, and unlike ASA 3, we prefer to administer fentanyl, which supports hemodynamic stability better than morphine. Higher ASA classification increases the risk of DHR and can also be more fatal [8]. A Singaporean study also found that higher ASA classification was associated with a higher risk for time to develop DHR [9].

#### History of allergy

Out of all the different types of food, we found that seafood allergy increased the risk of perioperative DHR event (risk score of 2) when compared to not having any food allergy. Seafood, especially fish, allergy, was shown to be highly associated with intraoperative DHR [8,21]. If a patient has a fish allergy, especially to salmon, then they are likely to be allergic to protamine, since protamine is extracted from the sperm or mature testis of fish [8,12,22]. A previous study done in Thailand also found that the most common cause of anaphylaxis was food allergy, particularly seafood allergy [23,24].

#### Antibiotics

The usual practice in our institution is for the surgeon to ask the anesthesiologist to administer antibiotics within 1 hour before commencing surgery. We initially give a small volume of diluted antibiotics (test dose) and wait to see if there is any allergic reaction before administering the complete volume. If an allergic reaction occurs during administration of the test dose, the surgeon, after being notified, will decide perioperatively which alternative antibiotic (if any) to administer. However, we found that cefazolin and ceftriaxone increased the risk of perioperative DHR (risk score of 1), a result which was supported by many other studies [11,25]. In our practice, ceftriazone (a beta-lactam) is the second most commonly used antibiotic during the perioperative period and clindamycin is used when the patient has a history of beta-lactam reaction.

#### Narcotics

Narcotics (morphine and pethidine) increased the risk of DHR in our study (Table 5). Morphine, together with fentanyl, is one of the most common narcotic agents used in our institute during general anesthesia. Pethidine was an intermediate risk factor of developing DHR (risk score of 3.0). In our institute, pethidine is not used as the main narcotic but reserved for treating shivering after neuraxial block during the intraoperative period or during recovery at the post-anesthetic care unit. Analgesic allergy can cause delayed minor DHR like urticaria and/or angioedema and also delayed type hypersensitivity rashes. Pfützner et.al [26] reported that analgesics are one of the elicitors of perioperative DHR which supports our finding. Perioperative use of opioids leading to perioperative DHR is rare [27,28]. DHR caused by pethidine could be due to non-specific histamine release and might not be an IgE-mediated reaction [29].

#### Protective factors of perioperative DHR

We found that volatile anesthetics reduces the risk of perioperative DHR (risk score: sevoflurane of -2.5 and desflurane of -2.0). There is no published report of anaphylaxis to halogenated volatile anesthetics [29], thereby use of volatile anesthetics to achieve adequate depth of anesthesia avoids the use of intravenous anesthetics, which reduced the risk of DHR in our study. Reports of local anesthetic hypersensitivity reactions were very rare (incidence of <1%) [30]. We found that levobupivacaine, but not bupivacaine, reduced the risk of perioperative DHR (risk score of -3.5). Levobupivacaine allows stereoselective binding of sodium and potassium channels and demonstrated less affinity and strength of inhibitory effect than the racemic parent or dextrobupivacaine and consequently decreased the toxicity profile [31,32].

### Subgroup analysis for moderate to severe DHR

#### High risk group

History of allergic rhinitis entailed a high risk of developing moderate to severe DHR (risk score of 5) but not overall DHR in our study. Other studies suggest that atopic disease is a risk factor for anaphylaxis, which may be triggered by food, drug or latex intake [4,5]. Patients who had a history of rhinitis may develop moderate to severe DHR which is triggered by different types of anesthetic agents.

#### Intermediate risk group

Increased duration of anesthesia for more than 2 hours entailed an intermediate risk of moderate to severe DHR (risk score of 1.5). Increased duration of anesthesia may expose the patient to an increased variety of anesthetic agents (opioids and NMBA) and antibiotics.

#### Clinical implication in anesthesia practice

Even though our predictive tool for perioperative DHR provided a modest predictive ability (AUC = 0.68), the predictive tool for moderate to severe DHR provided high specificity (80%). The tool may be used to reduce the risk of moderate to severe DHR perioperatively and can be applied in our practice. Patients will be classified into the high-risk group (risk score ≥2.5) for developing moderate to severe DHR if they have any history of drug allergy (risk score of 3.5 to 4.0) or rhinitis (risk score of 5). The risk would be greater if they receive intraoperative morphine (risk score of 1.0) or if their duration of anesthesia exceeds 2 hours (risk score of 1.5). Our study constructed a predictive tool of moderate to severe DHR in which only 10 patients had grade 3 DHR (severe life-threatening multiorgan involvement). Thus, the risk score might predict moderate DHR better than severe DHR. To predict a risk score of pure grade 3 DHR, a larger sample size may be required. However, using our predictive tool of at least grade 2 DHR (moderate multiorgan involvement) may prevent severe life-threatening multiorgan involvement.

For overall perioperative DHR, patients will be classified as high-risk (risk score ≥3.5) if they have a history of seafood allergy (risk score of 2.0) or drug allergy (risk score of 2.5), both patient-related factors, and if their airway is managed with endotracheal intubation (risk score of 3.5), if they received perioperative succinylcholine (risk score of 1.5) during intubation, opioids (except fentanyl, risk score of 1.0–3.0), or cephalosporin (risk score of 1.0). Therefore, morphine/pethidine, succinylcholine, and cephalosporin should be used with caution in patients who have a history of allergy. However, to reduce the risk of perioperative DHR, based on our risk predictive tool, one should apply inhalation anesthesia (risk score of -2.0 to -2.5) combined with local anesthetics, especially levobupivacaine (risk score of -3.5).

### Study strengths and limitations

The strengths of our study are as follows. First, the sample size was adequate under 90% power to avoid type 2 error. Second, the time to first DHR event and period of anesthesia were recorded by two independent anesthesiologists to reduce any possible misclassification of cases. Third, cases and controls were matched to reduce the selection bias by age and type of surgery thus minimizing the possibility of confounding. Fourth, the large sample size allowed us to perform subgroup analyses to determine if any of the risk factors identified from the overall results differed based on the severity of the DHR event. Despite these strengths, our study had some limitations. First, DHR was diagnosed clinically with no laboratory confirmation since the serum tryptase test could not be performed in our institute. Some patients could also not afford the cost of the skin test. Second, a retrospective study could lead to some information bias or misclassification of cases. Finally, patients were selected from a single center, thus generalizability of the results is limited.

## Conclusions

Our predictive tool for perioperative DHR provided a modest predictive ability whereas for moderate to severe DHR it provided high specificity but low sensitivity. Drug allergies and morphine use were the common risk factors. Endotracheal tube intubation was a strong risk factor of perioperative DHR whereas history of allergic rhinitis was a strong risk factor of moderate to severe DHR. If feasible, inhalation anesthesia combined with local anesthetics should be used to lessen the risk of a patient experiencing a hypersensitivity reaction.

## Supporting information

S1 Table Subgroup analysis of moderate/severe hypersensitive drug reaction among patient, surgery and anesthesia related factors. *p<0.05 by Fisher exact test, **p<0.05 by Chi-squares test, ***p<0.05 by Wilcoxon ranksum test. remote = cardiac catheterization/X-ray/gastrointestinal scope. Abbreviations: IQR, interquartile range; OPD, outpatient department; COPD, chronic obstructive pulmonary disease; ATB, antibiotics; Obgyn, obstetrics & gynecology; Ortho, orthopedics; Neuro, neurology; GU, genitourinary; ENT, ear-nose-throat; ASA, American Society of Anesthesiologists; GA, general anesthesia; RA, regional anesthesia; LMA, laryngeal mask airway; ETT, endotracheal tube intubation; PNB, peripheral nerve block.
(DOCX)

S2 Table Subgroup analysis of moderate/severe hypersensitive drug reaction among categories of anesthetic agent receiving during intraoperative period. **p<0.05 by Chi-squares test. NSAIDs, Nonsteroidal anti-inflammatory drugs.
(DOCX)

S1 File DHR Maliwan. (CSV)
