# Supplementary material for: Risk predictive tools of perioperative drug hypersensitivity reaction: A case-control study
Source: PLoS One. 2022 Jan 13;17(1):e0262362. doi: 10.1371/journal.pone.0262362 (PMC8758003; doi:10.1371/journal.pone.0262362)
Supplement: S1 Table — *p<0.05 by Fisher exact test, **p<0.05 by Chi-squares test, ***p<0.05 by Wilcoxon ranksum test. remote = cardiac catheterization/X-ray/gastrointestinal scope. Abbreviations: IQR, interquartile range; OPD, outpatient department; COPD, chronic obstructive pulmonary disease; ATB, antibiotics; Obgyn, obstetrics & gynecology; Ortho, orthopedics; Neuro, neurology; GU, genitourinary; ENT, ear-nose-throat; ASA, American Society of Anesthesiologists; GA, general anesthesia; RA, regional anesthesia; LMA, laryngeal mask airway; ETT, endotracheal tube intubation; PNB, peripheral nerve block. (DOCX) [file pone.0262362.s001.docx]

## S1 Table. Subgroup analysis of moderate/ severe hypersensitive drug reaction among patient, surgery and anesthesia related factors

| Variable | Mod/severe HDR  (n= 88) | Control (n=176) | p-value |
| --- | --- | --- | --- |
| Patient related factors |  |  |  |
| Age, mean [SD], years | 39.8 (19.9) | 41.2 (20.3) | 0.592 |
| Age group, years: 0-1/ 1-7/ 8-18/ 19-65/ >65 | 1/ 4/ 8/ 64/ 11 | 2/ 8/ 16/ 128/ 22 | Match |
| Male/ Female | 32/ 56 | 72/ 104 | 0.563 |
| Case (OPD/ Elective/ Emergency) | 1/ 70/ 17 | 2/ 131/ 43 | 0.748 |
| Concomitant disease |  |  |  |
| Asthma (Y/ N) | 1/ 87 | 1/ 175 | 1 |
| Allergic Rhinitis (Y/ N) | 8/ 80 | 1/ 175 | <0.001* |
| COPD (Y/ N) | 0/ 88 | 1/ 175 | 1 |
| Hypertension (Y/ N) | 14/ 74 | 33/ 143 | 0.69 |
| History of allergy |  |  |  |
| Drugs (Y/ N) | 19/ 69 | 11/ 165 | 0.001****** |
| At least 2 categories/ ATB/ Analgesic/ Others | 7/ 4/ 5/ 3 | 2/ 3/ 2/ 4 | 0.002* |
| Foods (Y/ N) | 6/ 82 | 6/ 170 | 0.223 |
| Seafood/ Daily product | 5/ 1 | 4/ 2 | 0.30 |
| Surgery and anesthesia related factors |  |  |  |
| Type of Surgery |  |  | Match |
| Cardiac/ Obgyn/ Ortho/ Neuro/ GU/ ENT/ Vascular/ Plastic/ Abdomen/ Thoracic/ Remote/ Eye | 2/ 26/ 22/ 5/ 6/ 6/ 2/ 1/ 8/ 3/ 3/ 1 | 4/ 52/ 44/ 10/ 12/ 12/ 4/ 2/ 16/ 6/ 6**/** 2 |  |
| ASA physical status (1/ 2/ 3/ 4) | 9/ 60/ 18/ 1 | 13/ 105/ 55/ 3 | 0.249 |
| Type of anesthesia (GA only/ RA only/ Combined GARA) | 60/ 16/ 12 | 120/ 30/ 26 | 0.953 |
| Airway management (Room air/ oxygen insufflation / Mask/ LMA/ ETT) | 12/ 0/ 0/ 8/ 68 | 26/ 5/ 2/ 10/ 133 | 0.408 |
| Type of RA (Spinal block only/ PNB only/ Epidural block only/ Combined spinal and PNB) | 12/ 11/ 4/ 1 | 25/ 20/ 9/ 2 | 0.995 |
| Duration of anesthesia, median [IQR], min | 175 [120, 287.5] | 150 [90, 252.5] | 0.032*** |
| Duration of anesthesia, hours (<2/ ≥2) | 21/ 67 | 70/ 106 | 0.015** |
| Estimate blood loss at the time of symptom, median [IQR], ml | 175 [37.5, 400] | 100 [30, 400] | 0.402 |

*p<0.05 by Fisher exact test, **p<0.05 by Chi-squares test, ***p<0.05 by Wilcoxon ranksum test. remote= cardiac catheterization/ X-ray/ gastrointestinal scope. Abbreviations: IQR, interquartile range; OPD, outpatient department; COPD, chronic obstructive pulmonary disease; ATB, antibiotics; Obgyn, obstetrics & gynecology; Ortho, orthopedics; Neuro, neurology; GU, genitourinary; ENT, ear-nose-throat; ASA, American Society of Anesthesiologists; GA, general anesthesia; RA, regional anesthesia; LMA, laryngeal mask airway; ETT, endotracheal tube intubation; PNB, peripheral nerve block.
