# Supplementary material for: Risk predictive tools of perioperative drug hypersensitivity reaction: A case-control study
Source: PLoS One. 2022 Jan 13;17(1):e0262362. doi: 10.1371/journal.pone.0262362 (PMC8758003; doi:10.1371/journal.pone.0262362)
Supplement: S2 Table — **p<0.05 by Chi-squares test. NSAIDs, Nonsteroidal anti-inflammatory drugs. (DOCX) [file pone.0262362.s002.docx]

## S2 Table. Subgroup analysis of moderate/ severe hypersensitive drug reaction among categories of anesthetic agent receiving during intraoperative period

| Variable | Mod/severe HDR  (n= 88) | Control (n=176) | p-value |
| --- | --- | --- | --- |
| Intravenous induction agents (Y/N) | 77/11 | 149/27 | 0.664 |
| Propofol (Y/N) | 74/14 | 144/32 | 0.774 |
| Thiopental (Y/N) | 0/88 | 1/175 | 1 |
| Etomidate (Y/N) | 0/88 | 2/174 | 0.554 |
| Ketamine (Y/N) | 4/84 | 5/171 | 0.487 |
| Narcotics (Y/N) | 87/1 | 168/8 | 0.279 |
| Fentanyl (Y/ N) | 66/22 | 134/42 | 0.96 |
| Morphine (Y/ N) | 41/47 | 57/119 | 0.034* |
| Pethidine (Y/ N) | 2/86 | 1/175 | 0.258 |
| Neuromuscular blocking agents (Y/N) | 67/21 | 134/42 | 1 |
| Cisatracurium (Y/ N) | 52/36 | 106/70 | 0.965 |
| Rocuronium (Y/ N) | 16/72 | 27/149 | 0.68 |
| Succinylcholine (Y/ N) | 5/83 | 13/163 | 0.796 |
| Sedative agents (Y/N) | 26/62 | 49/127 | 0.885 |
| Midazolam (Y/ N) | 26/62 | 49/127 | 0.885 |
| Dexmedetomidine (Y/N) | 0/232 | 0/646 | - |
| Inhalation anesthetic agents (Y/N) | 62/26 | 137/39 | 0.245 |
| Sevoflurane (Y/N) | 57/31 | 126/50 | 0.322 |
| Desflurane (Y/N) | 5/83 | 11/165 | 1 |
| Antibiotics (Y/N) | 289/36 | 146/30 | 0.953 |
| Ampicillin (Y/N) | 6/82 | 12/164 | 1 |
| Cefazolin (Y/N) | 55/33 | 101/75 | 0.507 |
| Ceftriazone (Y/N) | 9/79 | 18/158 | 1 |
| Metronidazone (Y/N) | 4/84 | 11/165 | 0. 778 |
| Clindamycin (Y/N) | 5/83 | 4/172 | 0.165 |
| Others (Y/N) | 7/81 | 16//160 | 0.98 |
| NSAIDs (Y/N) | 7/81 | 15/161 | 1 |
| Ketorolac (Y/N) | 1/87 | 2/174 | 1 |
| Dynastat (Y/N) | 6/82 | 13/163 | 1 |
| Reversal agents (Y/N) | 52/36 | 103/73 | 1 |
| Neostagmine (Y/N) | 50/38 | 102/74 | 0.965 |
| Suggamadex (Y/N) | 2/86 | 1/175 | 0.258 |
| Anticholinergic (Y/N) | 50/38 | 101/75 | 1 |
| Atropine (Y/N) | 50/38 | 101/75 | 1 |
| Glycopyrrolate (Y/N) | 0/88 | 0/176 | - |
| Regional anesthesia (Y/N) | 30 /58 | 49/127 | 0.367 |
| Lidocaine (Y/N) | 8/80 | 7/169 | 0.159 |
| Hyperbaric bupivacaine (Y/N) | 9/79 | 25/151 | 0.475 |
| Isobaric bupivacaine (Y/N) | 15/73 | 16/160 | 0.091 |
| Levobupivacaine (Y/N) | 0/88 | 5/171 | 0.173 |

**p<0.05 by Chi-squares test. NSAIDs, Nonsteroidal anti-inflammatory drugs.
